# Role of Carbon, Nitrogen, Phosphate and Sulfur Metabolism in Secondary Metabolism Precursor Supply in *Streptomyces* spp.

**DOI:** 10.3390/microorganisms12081571

**Published:** 2024-07-31

**Authors:** Sergii Krysenko, Wolfgang Wohlleben

**Affiliations:** 1 Department of Microbiology/Biotechnology, Interfaculty Institute of Microbiology and Infection Medicine Tübingen (IMIT), University of Tübingen, Auf der Morgenstelle 28, 72076 Tübingen, Germany; sergii.krysenko@uni-tuebingen.de; 2 Cluster of Excellence ‘Controlling Microbes to Fight Infections’, University of Tübingen, 72076 Tübingen, Germany; 3 German Center for Infection Research (DZIF), Partner Site Tübingen, 72076 Tübingen, Germany

**Keywords:** primary metabolism, secondary metabolism, *Streptomyces*, bacterial physiology, precursor supply

## Abstract

The natural soil environment of *Streptomyces* is characterized by variations in the availability of nitrogen, carbon, phosphate and sulfur, leading to complex primary and secondary metabolisms. Their remarkable ability to adapt to fluctuating nutrient conditions is possible through the utilization of a large amount of substrates by diverse intracellular and extracellular enzymes. Thus, *Streptomyces* fulfill an important ecological role in soil environments, metabolizing the remains of other organisms. In order to survive under changing conditions in their natural habitats, they have the possibility to fall back on specialized enzymes to utilize diverse nutrients and supply compounds from primary metabolism as precursors for secondary metabolite production. We aimed to summarize the knowledge on the C-, N-, P- and S-metabolisms in the genus *Streptomyces* as a source of building blocks for the production of antibiotics and other relevant compounds.

## 1. Actinobacteria and Their Primary Metabolism as Basis for Secondary Metabolism Precursor Supply

The Actinomycetota (Actinobacteria or Actinomycetes) are a phylum containing a group of terrestrial and aquatic Gram-positive bacteria with a high G + C content. This phylum includes bacteria that can be isolated from many different environments. Bacteria from this group are involved in the decomposition of organic materials like chitin and cellulose and play an important role in the nitrogen and carbon cycles as well as in organic matter turnover in nature. In comparison to fungi, Actinomycetes are much smaller and occupy many shared ecological niches, but also specific ones. They replenish the supply of nutrients in the soil that is a vital part of humus formation.

Actinomycetes include following representative genera: *Actinomyces*, *Arthrobacter*, *Corynebacterium*, *Frankia*, *Micrococcus*, *Micromonospora*, *Mycobacterium*, *Nocardia*, *Propionibacterium* and *Streptomyces* [1]. Some soil Actinobacteria, such as Frankia, are endosymbionts of plants that are able to fix nitrogen in exchange for access to saccharides and other nutrients from the plant. Other species, such as some members of the genera *Mycobacterium*, *Corynebacterium*, *Nocardia* and *Rhodococcus*, are human pathogens.

More than two-thirds of all known antibiotics are synthesized by Actinobacteria from the genus *Streptomyces* [2], and there are many more unknown compounds produced by *Streptomyces* spp. Recent studies showed that bacteria from this genus can synthesize more than 150,000 antimicrobial compounds that are currently unknown [3]. *Streptomyces* possess mechanisms to govern the metabolic pathways involved in production of secondary metabolites in response to nutrient availability and other external signals. Diverse substrates can induce the production of antibiotics, which may involve the activation of antibiotic-producing enzymes as well as the regulation of the biosynthetic activity of such enzymes. Nutrients like nitrogen-, carbon- and phosphate-containing compounds were reported to indirectly modulate antibiotic production by influencing the primary metabolism that provides precursor molecules for secondary metabolite biosynthesis [4]. The feedback and feedforward regulation coupled with the regulation of the nutrient supply have been shown to be mechanisms that could be applicable for the industrial production of secondary metabolites [5].

### 1.1. Carbon Metabolism as a Source of Precursors

Actinobacteria live in soil under varied nutrient conditions and therefore are able to utilize different C-sources, including monosaccharides, polyols, disaccharides, amino acids, sugar alcohols, deoxy sugars, glycosides, dicarboxylic acids, ketoacids [6,7], polyamines and monoamines including ethanolamine [8].

From these substrates, hexoses are preferred because they can be directly introduced into glycolysis. Many other substrates require multiple catalytic reactions in order to allow the incorporation of such compounds into cellular metabolism. This requires additional energy, and therefore, processes are necessary to prioritize energy usage. The cells should be constantly protected against wasting the protein-synthesizing machinery. A mechanism to ensure this is carbon catabolite repression (CCR), which is an important part of the global control system in bacteria. It has been extensively studied in *E. coli*, but also investigated as a conserved mechanism in high GC Gram-positive bacteria including *Streptomyces* spp. and *Mycobacterium* spp. [9,10]. The selection of preferred carbon sources is especially important and is considered to be a main determining factor in the microbial growth rate, supporting successful competition against other microorganisms in the environment [11]. The most optimal carbon source is glucose. Used in high concentrations, it interferes with the formation of diverse compounds in primary and secondary metabolisms. CCR allows for the repression of the protein synthesis due to the presence of a catabolite that is generated from a rapidly metabolizable exogenous carbon source. In *S. coelicolor*, cAMP does not appear to play a role in CCR because cAMP levels do not vary [6,9,12].

*Streptomyces* were shown to utilize unique CCR mechanisms to modulate the rates of carbon consumption that affect the expression of genes involved in the transport of alternative carbon sources, e.g., glycerol and arabinose, xylose, fructose and galactose [10]. Furthermore, CCR affects the production of extracellular polysaccharide-degrading enzymes, such as chitinase and amylase [13]. Carbon source availability has been demonstrated to influence antibiotic production, enzyme secretion and morphological development in streptomycetes [14].

**Figure 1:**
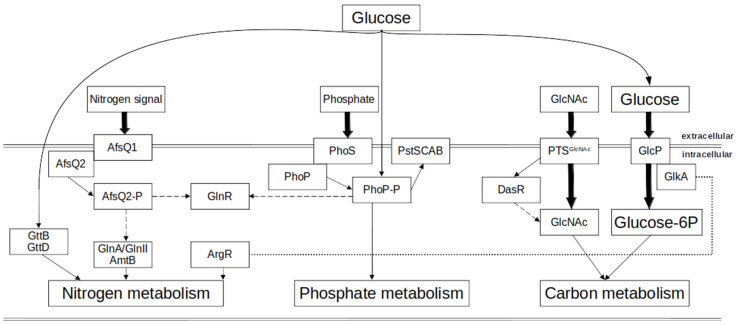
A scheme of the main regulatory interconnections of carbon, nitrogen and phosphate metabolisms in *Streptomyces coelicolor*. Arrows indicate positive effects, while dashed arrows indicate negative effects. The dotted lines indicate an uncharacterized or indirect regulation (modified from [25,26]).

The main carbon source in *Streptomyces* spp. is glucose. It is predominantly transported through the GlcP transporter belonging to the MFS family [12,15]. Subsequently, it is phosphorylated by the glucose kinase GlkA, resulting in the formation of glucose 6-phosphate (Figure 1). In *Streptomyces*, GlkA is involved in the global regulation of CCR. Glucose kinase shares identity with proteins from the ROK family (“Repressor, ORF, Kinase”, a family of bacterial proteins including transcriptional repressors, sugar kinases and uncharacterized open reading frames). ROK proteins were demonstrated to have regulatory functions in *Streptomyces* spp., e.g., in *S. avemitilis* [16]. Glucose kinase combines features of transcriptional regulators (a helix-turn-helix DNA-binding domain) and of kinases. Interestingly, in *S. coelicolor,* GlkA lacks the DNA-binding domain and it cannot exert a regulatory function by binding to the promoter regions of the repressed genes. It has been demonstrated that GlkA interacts with GlcP in the efficient transport and phosphorylation of glucose [17]. Recent studies demonstrated that CCR functions through two mechanisms in *S. coelicolor*: the GlkA-dependent and glucose-dependent mechanisms [18]. It has been shown that glucose-stimulated glycolysis and the pentose phosphate pathway negatively affect the enzymes involved the amino acid metabolism. Furthermore, a strong influence of GlkA on the synthesis of prodigiosins (RED) and calcium-dependent antibiotics (CDAs) was observed [18,19]. Recent studies showed that the CCR effect is based on the combined action of GlkA and transcription factors [18]. Although Glk plays a role in CCR, glucose also play a key role in this mechanism. Transcriptome studies demonstrated that the expression of 40 different DNA binding proteins is regulated by glucose. These include 31 transcriptional regulators as well as 4 two-component systems. Such proteins are involved in the signals elicited by glucose [18]. The transcriptional factors stimulated by glucose include transcriptional factors from the TetR, MerR, GntR, LacI, MarR and AsnC families and sigma factors. The transcriptional factors repressed by glucose include members of the DeoR, GntR and MarR families as well as sigma and anti-sigma factors [18,20]. The terminal step of glycolysis is catalyzed by pyruvate kinase (PK), which converts phosphoenolpyruvate to pyruvate using ADP as the phosphor acceptor. This reaction results in the production of ATP. PK plays a key role in linking glycolysis and the citric acid cycle [21].

Some studies reported that in *S. tsukubaensis*, the utilization of glucose as a carbon source promotes the expression of genes of the Pho regulon *phoR*–*phoP* and *pstS*, and that glucose significantly stimulates the genes involved in phosphate transport [22]. Also, glycerol and other carbon sources were shown to influence the expression of genes involved in phosphate metabolism [23,24]. The coordination of carbon and phosphate utilization through the glycolysis was shown to play an important role in the metabolism of *Streptomyces* [5].

### 1.2. Primary Metabolism in Actinobacteria: Nitrogen Metabolism

#### 1.2.1. Biochemistry of Nitrogen Metabolism

Actinobacteria use a variety of nitrogen sources for growth, including simple compounds like ammonium, nitrate and nitrite [6] as well as complex organic sources of nitrogen like amino acids (e.g., glutamate, glutamine, histidine and arginine) [27,28], urea, amino sugars, peptones [29] and other nitrogen-containing compounds like monoamines and polyamines [8]. Furthermore, Actinobacteria from the genus *Frankia* are able to utilize atmospheric nitrogen for the production of organic nitrogen compounds [30]. Generally, ammonium is the preferred nitrogen source. It represses the use of other nitrogen sources, e.g., nitrate [31]. Under high concentrations, ammonium can also inhibit the growth of most Actinobacteria [32]. The diverse nitrogen sources are converted to glutamate and/or glutamine. These amino acids serve as amino donors, mostly in transamination reactions to form other nitrogen-containing compounds.

Most Actinobacteria possess a functional glutamine synthetase, GlnA (also referred as GlnA1), that catalyzes glutamine synthesis from ammonium and glutamate under low nitrogen concentration conditions. Species of some genera, e.g., *Streptomyces*, have two glutamine synthetases, encoded by the genes *glnA* and *glnII*. Both glutamine synthetases GlnA and GlnII convert glutamate to glutamine, while the major glutamine synthetase activity comes from GlnA [33]. GlnII was first identified in *Rhizobium*. It was hypothesized that GSII may have been obtained by gamma-Proteobacteria from plants through horizontal gene transfer at an early stage of evolution. But, further studies demonstrated that GSII originated from bacteria [34]. Plants seemed to have acquired GSII via chloroplasts. GSII has been found in many representatives of the Actinomycetales, but it is absent in *Amycolatopsis*, *Mycobacterium* and *Corynebacterium* [34,35,36,37].

Interestingly, glutamine synthetase-like enzymes (GlnA2, GlnA3 and GlnA4) have been reported in *Streptomyces* [37,38,39] and *Mycobacteria* [40], but also in other actinobacterial genera, like *Frankia*, *Microbispora*, *Rhodococcus* and *Brevibacterium.* These enzymes do not show GS activity; instead, GlnA2 and GlnA3 are gamma-glutamylpolyamine synthetases [38] and GlnA4 is a gamma-glutamylethanolamide synthetase [39].

**Figure 2:**
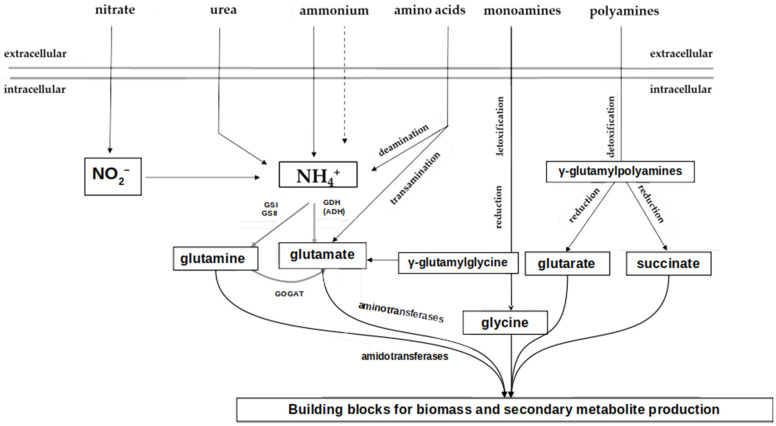
Schematic representation of primary nitrogen metabolism in *Streptomyces coelicolor*. Black lines represent transport through the cell membrane as well as enzymatic reactions in the cell; dashed line—ammonium diffusion through the cell membrane; grey lines represent the cell membrane. GS, glutamine synthetase; GDH, glutamate dehydrogenase; GOGAT, glutamine-2-oxoglutarate-aminotransferase (modified from [43]).

While GS is preferably active at low nitrogen concentrations, ammonium is metabolized by glutamate dehydrogenase (GDH) under high ammonium concentration conditions. Some Actinomycetales contain two GDH enzymes that catalyze the conversion of ammonium to glutamate (reductive amination) and the reverse reaction. The glutamate cleavage (oxidative deamination) during ammonium dissimilation is carried out by a catabolic, NADH-dependent glutamate dehydrogenase [41]. GSI and assimilatory GDH do not function side by side in all bacteria. In some other species, e.g., in *S. hygroscopicus* and *S. clavuligerus*, alanine dehydrogenase (ADH) is active instead of GDH in ammonium assimilation. ADH catalyzes an amination of pyruvate that leads to alanine formation [41,42] (Figure 2).

#### 1.2.2. Regulation of Nitrogen Metabolism in Actinobacteria

The regulation of nitrogen metabolism takes place at the transcriptional and the post-translational levels. This regulation in Actinomycetales is distinct from that in Enterobacteriaceae.

Several transcriptional regulatory components known from Enterobacteriaceae, such as GlnK (=P_II_), GlnD, GlnE and Crp have counterparts in Actinomycetales, but their individual function and interplay are different. Regulators with functionality specific to Actinobacteria like GlnR, GlnRII and NnaR are only present in some actinomycetes like *Streptomyces*. The complex nitrogen metabolism of *S. coelicolor* requires additional control by other transcriptional regulators: ArgR, AfsR, AfsQ1, DasR and PhoP [44,45,46] (Figure 1). Interestingly, it has been demonstrated that *glnA2*, *glnA3* and *glnA4* in *S. coelicolor* are regulated differently at the transcriptional level. A strong interaction between acetylated GlnR and the *glnA2* promoter region has been revealed. *glnA3* and *glnA4* promoter regions appeared to interact with specialized regulators EpuRII and EpuRI, respectively [39].

**Figure 3:**
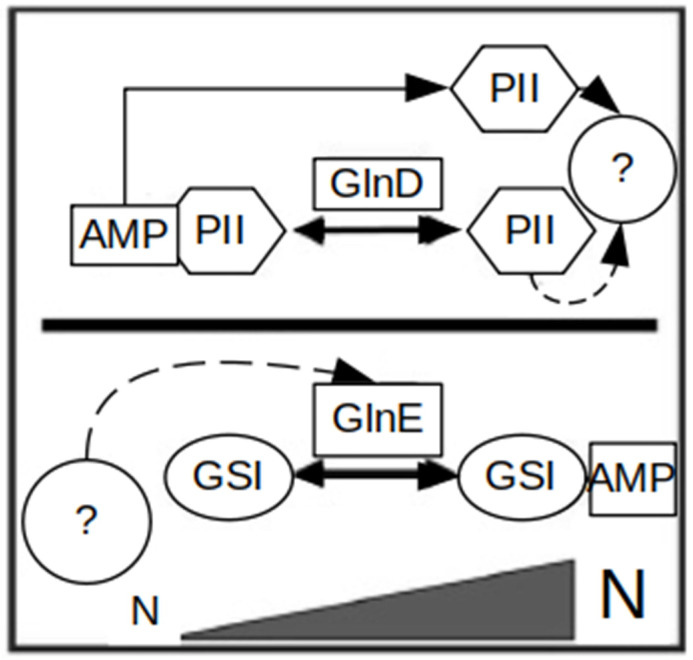
Scheme of the post-translational regulation of the GS enzyme in *S. coelicolor*. The activity of GlnA (GSI) is regulated through reversible adenylation and deadenylation by an adenylyltransferase (GlnE). In contrast to *E. coli*, in *S. coelicolor*, the PII protein as well as the adenylyltransferase GlnD are not essential for the GlnE-dependent GSI regulation. Straight arrows represent confirmed interactions, dashed lines—unstudied. AMP, adenosine monophosphate; GSI, glutamine synthetase I; UMP, uridine monophosphate; question mark, unknown regulatory protein (modified from [8]).

At the post-translational level, GSI is controlled by GlnE via adenylation and deadenylation [33] (Figure 3). Interestingly, GSII lacks post-translational regulation through adenylation [36]. Also, the GS-like enzymes GlnA2, GlnA3 and GlnA4 lack an adenylation motif and cannot be modulated post-translationally by adenylation like GSs.

### 1.3. Primary Metabolism in Actinobacteria: Phosphate Metabolism

#### 1.3.1. Biochemistry of Phosphate Metabolism

Another essential nutrient element required for metabolism in Actinobacteria is phosphate. Its deficiency causes the down-regulation of primary metabolism. On the other hand, it can trigger the production of secondary metabolites [47]. Phosphorous is present in nature in the form of phosphate salts, organophosphates and phosphonates. Actinobacteria are able to transport inorganic phosphate using the phosphate transport system PstSCAB as well as the PitH transporters. The PstSCAB system has a higher affinity and consists of four components [48].

In *Streptomyces* spp., PstS (phosphate-binding protein) is attached to the outer side of the cell membrane, glycosylated and released. The transport of phosphate is strictly regulated by the PstSCAB system through the concentration of inorganic phosphate and mediated by the binding of phosphorylated PhoP to the promoter region of the PstSCAB operon [48]. Some Actinobacteria, e.g., *Mycobacterium smegmatis*, have an additional transport system with high-affinity PhnCDE, which is also regulated by PhoP. Furthermore, *Streptomyces* spp. have a phosphate transport system with a low affinity, encoded by the *pitH1*–*pitH2* genes. In *S. coelicolor*, PhoP regulates the expression of *pitH2*, but not that of *pitH1*. In *Streptomyces*, an increase in the concentration of hexoses can favor the uptake of inorganic phosphate to control carbon and phosphate metabolisms. This occurs through the pentose phosphate pathway, glycolysis and TCA cycle [49,50].

In many *Streptomyces* spp., the co-localized gene clusters *pitH1-pstSCAB-ppk* (polyphosphate kinase) are linked in a supercluster formed by nine genes related to phosphate metabolism. The *ppk* gene encodes an enzyme that catalyzes the reversible polymerization of the gamma phosphate of ATP into polyphosphate. It was shown to play a negative role in the control of antibiotic biosynthesis in *Streptomyces*. Nucleotides and sugar phosphates are used as phosphate sources by *Streptomyces* spp. too. They are dephosphorylated by extracellular phosphatases and nucleotidases. Furthermore, strains of *Arthrobacter* and *Streptomyces* were reported to be able to degrade simple phosphonates [48,51,52].

#### 1.3.2. Regulation of Phosphate Metabolism

Phosphate control in Actinobacteria has been studied in *Streptomyces*, in which it is mediated by the two-component system (TCS) PhoR–PhoP; its orthologs have been found in most Actinobacteria [53,54]. It belongs to class IIIA of TCSs with the PhoR protein acting as a sensor kinase [53,55]. PhoP belongs to the OmpR family of DNA-binding response regulators. The PhoR protein kinase can self-phosphorylate under phosphate depletion conditions. The phosphate group is then transferred to a receiver aspartate in PhoP. This causes a structural rearrangement and activation of the DNA-binding domain. The phosphorylated PhoP can activate or repress the expression of genes in the *Pho* regulon [23]. PhoR–PhoP has been shown to control the primary metabolism and secondary metabolism of *S. coelicolor, S. lividans*, *S. tsukubaensis, S. natalensis*, *S. avermitilis* and some others [22,24,25,52,56] (Figure 1).

Phospholipids (e.g., phosphatidylserine, phosphatidylglycerol, phosphatidylcholine and phosphatidylethanolamine) are present in all living organisms. They are abundant in some habitats as part of decaying plants and animals [48]. Glycerol phosphodiesters are synthesized by the deacylation of phospholipids. They keep a phosphate at carbon-3 of the glycerol backbone. The phosphodiesters can be hydrolyzed by glycerophosphodiester phosphodiesterases (GDPDs), which cleave the ester bond, leading to the release of glycerol-3-phosphate. Soil dwelling actinobacteria like *Streptomyces* are able to utilize the phosphate from glycerophosphodiesters. In *S. coelicolor*, seven putative *gdpg* genes have been characterized. Three of these genes are named *glpQ1* through *glpQ3*, encoding secreted GDPDs, and the other four genes encode intracellular uncharacterized phosphodiesterases. A gene orthologous to the *glpQ* genes of *S. coelicolor* can be found in the genomes of *S. avermitilis*, *S. clavuligerus* and *S. venezuelae*, and encode proteins with 67 to 80% amino acid identity. The PhoP regulator has been shown to regulate *glpQ1/glpQ2* in *Streptomyces*, which encode glycerophosphodiester phosphodiesterases [48,56,57].

### 1.4. Primary Metabolism in Actinobacteria: Sulfur Metabolism

Sulfur is a common compound in the environment (up to 0.1% of the earth’s crust). However, much of this material is inaccessible to living organisms. Sulfur is an essential element for bacterial metabolism and accounts for approx. 1% of the cell dry weight. It plays a central role in metabolic processes [58]. All bacteria require sulfur for growth. It is used in the biosynthesis of amino acids (e.g., cysteine and methionine), which are incorporated into other biomolecules [59]. Sulfur also plays an essential role in a variety of enzyme cofactors, such as coenzyme A, coenzyme M, biotin, thiamine and lipoic acid. Furthermore, sulfur is critical in redox processes as a building block for iron–sulfur centers and as the redox-active component of disulfide bonds. Furthermore, sulfane sulfur, including polysulfide and persulfide, was demonstrated to be a cellular component present in microorganisms, with reported impacts on the production of actinorhodin and spore formation in *S. coelicolor* M145 [60].

For biosynthetic processes, sulfur is generally derived from the assimilation of inorganic sulfate in bacteria and plants, but not in animals. In bacteria, sulfur amino acid (SAA) metabolism is highly adaptive and diversified [61,62]. In *Streptomyces* spp., the biosynthesis of cysteine (Cys) and methionine (Met) via homocysteine (Hcy) are linked through bidirectional transsulfuration pathways. Each transsulfuration direction involves two sequential reactions that are catalyzed by different enzymes that often have relaxed specificities. Cysteine is the key intermediate in most pathways of sulfur metabolism. After the inorganic sulfate is transported into the cell, it is incorporated into the sulfate assimilation pathway. Sulfur is converted to 3′-phosphoadenosine-5′-phosphosulfate (PAPS), and then reduced to sulfite and subsequently to sulfide. Afterwards, sulfide is transferred onto an organic moiety to yield molecules of cysteine [59,61,62].

The inter-conversion between homocysteine (Hcy) and methionine takes place in the activated methyl cycle (AMC), which involves ATP and additional cofactors such as cobalamin and 5-methyl-tetrahydrofolate. The function as well as metabolic branching of the AMC have been demonstrated to influence the production of bacterial metabolites (e.g., quorum-sensing molecules) that can have an impact on the bacterial physiology and the surrounding microbial community [62]. Metabolism of the sulfur-containing amino acids methionine and cysteine requires the transfer of methyl groups. Afterwards, these amino acids are converted to compounds that enter central metabolic pathways, with the following oxidation of the sulfur atom and formation of excretory forms of sulfur, as well as with the synthesis of other essential compounds. The transfer of sulfur from methionine to serine by transsulfuration to synthesize cysteine is critical for methionine catabolism and sulfur excretion. Most *Streptomyces* spp. and other actinobacteria do not have a MetA enzyme, which acylates the hydroxyl of homoserine with succinyl-CoA. Instead, a direct sulfhydrylation pathway involving *metX* and *metY* has been found in actinomycetes. *S. coelicolor* apparently lacks *metA* and *metXY.* Most actinobacterial genomes also do not have a canonical *metC* that encodes for a cystathionine-β-lyase; instead, they have a *malY* gene [58,59,60,61,62].

The knowledge of sulfur metabolism in antibiotic-producing streptomycetes is generally fragmentary. The assimilation of sulfate into methionine has only been described in the model streptomycete *S. coelicolor*. The sulfate assimilation cluster in *S. coelicolor* was shown to be structurally and functionally similar to that of *Corynebacterium.* The genes *sco6093*-*sco6102* were demonstrated to be involved in sulfate assimilation, with *sco6102* encoding for sulfite reductase and the *sco6094*-*sco6096* genes being involved in sulfate import. The gene *sco6101* was shown to be essential for the production of methionine [63].

## 2. Specialized Metabolic Pathways for Supply of Alternative Precursors for Secondary Metabolism

In addition to the standard reactions for the synthesis of cellular building blocks, which occur similarly in many bacteria, Actinobacteria (due to their lifestyle in the soil) have developed various special abilities that allow them to exploit unusual C and N sources and use them to create the building blocks for secondary metabolite synthesis. Compounds that can be directly absorbed and used include polyamines and ethanolamine as well as compounds such as xylans and cellulose.

### 2.1. Alternative Metabolism in Actinobacteria—Polyamine Metabolism

In nature, polyamine metabolism can provide nitrogen and carbon in environments with unfavorable conditions. Moreover, recently, polyamines were reported to directly impact secondary metabolism in *Streptomyces* [8]. The metabolism of polyamines is shaped by evolutionary processes, such as gene duplication, loss, fusion and horizontal gene transfer [64].

Polyamines are aliphatic polycations with polycarbon chains and multiple amino groups. Natural polyamines that are widely distributed in bacteria are putrescine, cadaverine, spermidine and spermine [65]. Polyamines have been demonstrated to play important roles in bacterial cells, influencing cellular growth, developmental processes and environmental stress responses. Polyamines are predominantly derived from the amino acids ornithine and methionine, but can be also synthesized from arginine and lysine as secondary sources [65]. In *S. coelicolor,* low concentrations of putrescine, cadaverine, spermidine and spermine (ca. 0.05–0.1 μmol/g) were detected after incubation in a complex medium. Furthermore, putrescine, spermidine and diaminopropane biosynthesis can occur in *S. coelicolor* in the late stationary phase, while cadaverine is synthesized under iron limitation conditions [66].

Polyamines are present in soil, where they are released after the decomposition of organic matter. The average polyamine concentrations per gram of humus were determined in an analysis of soils and different sources of humic acids. Putrescine ranged between 0.28 and 0.56 nmol/g, spermidine was between 0.23 and 0.62 nmol/g, and spermine was between 0.16 and 0.43 nmol/g [67]. Polyamines can be acquired from the extracellular environment and assimilated as a nitrogen and carbon source. In the Actinobacterium *S. coelicolor*, this occurs via the gamma-glutamylation pathway [8]. The initial step of polyamine utilization is catalyzed by GlnA2 and/or GlnA3 enzymes that are able to glutamylate the polyamines putrescine, cadaverine, spermidine and spermine [38].

**Figure 4:**
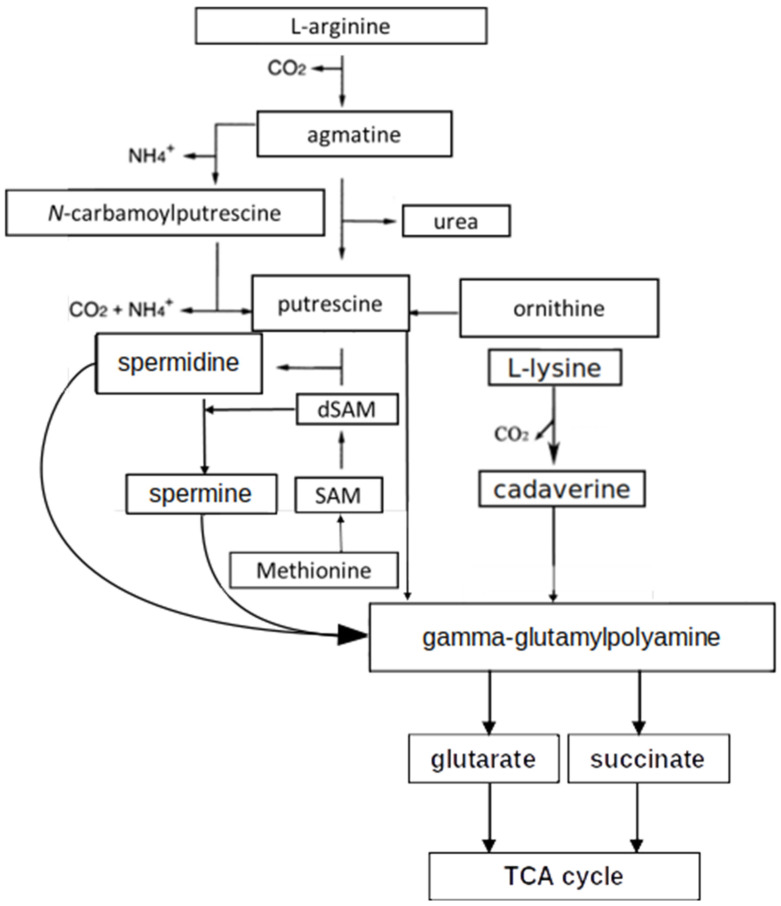
Combined scheme of polyamine biosynthetic and utilization pathways in *Streptomyces* (modified from [43,69]).

Sequence alignments and transcriptional analysis indicated that glutamylated products can be further metabolized to aminovalerate and GABA, a process involving a gamma-glutamylpolyamine oxidoreductase, dehydrogenases and hydrolases. Subsequently, a GABA aminotransferase catalyzes the production of glutarate semialdehyde or succinate semialdehyde (Figure 4). Succinic semialdehyde dehydrogenase converts these to succinate or glutarate that feeds the tricarboxylic acid (TCA) cycle [38]. Alternatively, in *S. coelicolor*, polyamines may be utilized via the aminotransferase pathway involving an amidotransferase.

Elevated polyamine concentrations can lead to bacterial cell death; for instance, 200 mM of exogenous putrescine was shown to be toxic to *S. coelicolor* [38]. Elevated polyamine levels occur in soil on sites with high amounts of decomposed organic matter. Thus, in the first step of the polyamine utilization pathway, polyamines are detoxified by glutamylation [38]. Besides their role as precursors, polyamines can influence secondary metabolism in a different way. In *S. coelicolor* grown in defined media with added exogenous polyamines as the sole nitrogen source, putrescine, cadaverine, spermidine and spermine caused delayed aerial mycelium development and spore formation. Furthermore, polyamines induced the production of prodigiosin and abolished the production of actinorhodin in *S. coelicolor* M145 [38]. Deletion of the essential gene for polyamine utilization, *glnA3 (sco6962)*, caused defects in aerial mycelium formation as well as sporulation in medium with glutamate as the nitrogen source. In media with nitrate, glutamine and ammonium as the nitrogen sources, increased actinorhodin and prodigiosin production was observed in the *S. coelicolor ΔglnA3* mutant [38,68]. In *S. tsukubaensis,* the presence of polyamines in the tacrolimus (FK-506) production medium resulted in inhibited biomass accumulation and FK-506 production. Poor growth of *S. tsukubaensis* was observed in media containing a high concentration of spermidine and spermine (25 mM). On the contrary, in *S. coelicolor*, up to 50 mM spermine and 100 mM spermidine in both complex and defined media did not inhibit the strain’s growth to the same extent as in *S. tsukubaensis* [68].

### 2.2. Alternative Metabolism in Actinobacteria—Ethanolamine Metabolism

A naturally occurring common monoamine is ethanolamine (or monoethanolamine), which is a primary amine and primary alcohol. It belongs to the class of aliphatic amino alcohols. In phospholipids, ethanolamine is a nitrogenous base. It is a building block of biomembranes along with glycerol, phosphoric acid and fatty acid esters [70]. Ethanolamine is a common component of cell membranes in the form of the phosphatidylethanolamine, which is the second most abundant head group for phospholipids. The incorporation of ethanolamine into cell membrane as well as its biological role have been studied in many Gram-positive bacteria such as *Mycobacterium* spp. [71], *Corynebacterium* spp., *Listeria* spp., *Enterococcus* spp. and *Clostridium* spp. [70,71,72] and recently in *S. coelicolor* [39], showing its importance for cellular homeostasis.

In Actinobacteria, the conversion of ethanolamine to glycine via a pathway with intermediates glycoaldehyde and glyoxylate was reported in *Mycobacterium* sp. 607 [71]. Reductive amination of glyoxylate to glycine by glycine dehydrogenase has been reported in *M. tuberculosis*. However, it was proposed that, in *Mycobacterium* spp., ethanolamine is not directly utilized as a C- or N-source as it is predominantly transformed into phosphatidylethanolamine, a component of cell membranes, through a pathway that includes ethanolamine phosphate and phosphatidylserine as intermediates (the process is also described as the biosynthetic utilization of ethanolamine) [71,72]. In *S. coelicolor*, the glutamylation of ethanolamine was confirmed recently and steps of the ethanolamine utilization pathway were postulated [39]. This pathway allows for the catabolism of ethanolamine as a C- and N-source when it is present in excess. Ethanolamine is glutamylated by GlnA4, a gamma-glutamylethanolamide synthetase. Afterwards, the pathway may require a predicted gamma-glutamylethanolamine dehydrogenase (SCO1611), a predicted gamma-glutamylaldehyde dehydrogenase (SCO1612) and a predicted gamma-glutamylglycine amidohydrolase (SCO1615). The pathway may end in the production of glycine and glutamate [39].

### 2.3. Exoenzymes and Proteases

*Streptomyces* typically act as saprophytes in soil. The nutrients found in this habitat are very diverse and many different polymers that can be used in primary and secondary metabolisms are present there. These include starch, cellulose, chitin, keratin, lignin, xylan and many others. Such compounds are mostly of plant origin, and therefore, the secretion of extracellular enzymes is required for the breakdown of these polymers, especially insoluble ones such as starch and cellulose. Soil environments are typically carbohydrate-rich but relatively deficient in nitrogen and phosphate [6,72].

In addition to taking up available building blocks or compounds that can be converted into building blocks, *Streptomyces* can rely on macromolecules that are broken down by exoenzymes, and whose fragments can then be picked up and processed as precursors. Their use of non-catalytic substrate-binding proteins and hydrolytic enzymes to obtain soluble nutrients from carbohydrates enables *Streptomyces* to interact with other organisms and promote cell growth. *Streptomyces* have a large repertoire of exoenzymes that have been extensively investigated and includes xylanases, proteases, cellulases, ligninases, proteases, chitinases, lipases, nucleases, keratinases and others [73,74,75]. Such extracellular enzymes are a collection of enzymes with a vital role in cell viability, stress responses and pathogenicity.

The insoluble nitrogen-containing polysaccharide chitin is a major nutrient source for streptomycetes. Chitinases have been isolated from various *Streptomyces* strains [73]; most species contain chitinase genes, enabling them to hydrolyze chitin. For instance, the *S. coelicolor* genome encodes 11 deduced family-18 chitinases and two family-19 chitinases. Furthermore, streptomycetes have a variety of extracellular enzymes for xylan hydrolysis [76] and can degrade carboxymethyl cellulose using secreted enzymes; 25 of the 180 *Streptomyces* strains can hydrolyze Avicel (microcrystalline cellulose). Most streptomycetes are saprophytic, but some phytopathogenic species like *S. scabies* are dependent on the inhibition of plant cellulose synthesis that is achieved though the production of thaxtomins and 4-nitroindol-3-yl-containing 2,5-dioxopiperazines [77]. *Streptomyces* spp. are large producers of proteases [78]. For example, in *S. coelicolor*, 21 genes code for aminopeptidases, 27 for serine proteases and 8 for metalloproteases [79]. Extracellular proteases are involved in assimilating extracellular proteinaceous nitrogen sources, such as keratin by using keratinases [80]. Some of the numerous secreted proteases participate in developmentally significant extracellular cascades, which lead to the cannibalization of the substrate mycelium biomass to support aerial growth and sporulation [72,78].

Numerous reviews are dedicated to the exoenzymes in *Streptomyces* (e.g., see [72]). In this paper, the focus is on the interconnection between the exoenzymes and secondary metabolism in *Streptomyces*, especially the supply of the main building blocks for secondary metabolism (N, C, P and S).

## 3. Utilization of Precursor Compounds from Primary Metabolism in Secondary Metabolism

### 3.1. Secondary Metabolism in Actinobacteria

In soil, nutrients are usually present in diverse complexity and abundance. Microorganisms need tools to sense, modify and use these compounds for biomass and secondary metabolite production. The soil is a complex and dynamic environment, and the activity of microorganisms plays a crucial role in multiple geochemical cycles. Secondary metabolites are not essential for the growth of the producer organisms, but they are important in various survival strategies [26,81].

Actinomycetota, which includes *Streptomyces*, is one of the largest bacterial groups participating in metabolic recycling in soil and the production of many antibiotics and other secondary metabolites such as antifungals, antivirals, antibacterials, immunomodifiers, enzyme inhibitors, antitumor drugs, insecticides, herbicides, etc. In *Streptomyces*, secondary metabolites are compounds that protect cells from environmental stresses and selective pressures [82]. For example, siderophores improve the absorption of iron from the environment; their biosynthesis is induced by iron deficiency in the cell, which can have a growth-promoting effect on the host organism [83]. Other examples are carotenoids, colored terpenoids that protect against photo-oxidative damage and oxygen radicals [84]. Other examples are pigments like melanin that can protect against UV damage [85]. Furthermore, another group of secondary metabolites is terpenoids, which can act as antibiotics, odorants, hormones and flavorings, e.g., a tricyclic sesquiterpene antibiotic albaflavenone [86] and ectoines that are effective against osmotic stress and are able to prevent protein misfolding [87]. Also, hopanoids were shown to be formed during the transition from substrate to aerial hyphae [88]. Other groups of compounds belonging to the group of antibiotics that can inhibit bacterial growth also represent a fitness advantage in the fight for nutrients. Secondary metabolites produced by *Streptomyces* can allow for the interaction with higher organisms. One of these well-studied symbioses is between the streptomycete *Streptomyces hygroscopicus* and the fungus *Aspergillus nidulans,* in which, secondary metabolite production is induced after direct physical contact between the two partners. The produced lecanor acid inhibits ATP biosynthesis and induces a symbiosis between the fungus and streptomycete [89]. Actinomycetes-derived antibiotics important for medicine include tetracyclines, aminoglycosides, macrolides, anthracyclines, glycopeptides, etc. [53,90].

The production of antibiotics is a very complex process involving different special genes, which are mostly located in gene clusters. In addition to the genes for the actual biosynthesis, genes for the transport, regulation and mediation of resistance are often included [91]. Due to the chemical structure and different modes of action, antibiotics can be can be divided into different classes according to biological activity: anti-infective antibiotics (antibacterial, antifungal and antiviral), cytostatics, herbicides, insecticides, acaricides and nematozoids. They can also be divided according to the chemical structure: lactams, aminoglycosides, macrolides, tetracyclines, lincosamides, sulfonamides, nitroimidazoles, polyketide antibiotics and polypeptide antibiotics. *Streptomyces* spp. are a large source of biologically active substances [2]. These include a variety of active compounds that are applied in numerous fields in medicine and agriculture. *Streptomyces* spp. produce antibacterials, antifungals, immunosuppressants and other bioactive compounds [92,93].

The secondary metabolites produced by Actinobacteria show a great variety, and their underlying chemical structure classes are also diverse, e.g., polyketides, polypeptides, lipoproteins, glycosides, terpenoids and alkaloids. Despite the great diversity, many natural products are based on similar biosynthetic principles. Basic units or preliminary stages are defined by a systematic mechanical, regulated production process to form a biologically active molecule [92]. There are various fundamentally similar synthesis methods for macromolecules of secondary metabolism, such as polyketide synthases (PKSs), NRPSs, aminoglycosides and others (e.g., RiPPs). Ribosomally synthesized and post-translationally modified peptides (RiPPs) are a superfamily of natural products that exhibits a range of structures, which begin as gene-encoded precursor peptides that are linear chains of amino acids produced by ribosomes. Polyketides are an important source of naturally occurring small molecules used for medicine. PKSs produce most of the commonly used antibiotics. Currently, three different mechanisms of polyketide biosynthesis are known and they are similar to the fatty acid biosynthesis mechanism. PKSs are classified into three types according to their biosynthesis mechanism: type I PKSs (modular type; non-iterative type), type II PKSs (iterative type), and type III PKSs (chalcone synthase type). Polyketide biosynthesis begins with an activated starter molecule, such as acetyl-CoA. The elongation of the molecule happens through activated extension units, usually malonyl-CoA. Different polyketide intermediate forms are possible. All polyketide biosynthetic mechanisms synthesize a basic body, which is then used for modifications or it serves as a precursor for further biosynthesis processes [94]. There are many other synthesis pathways, but their products do not produce such large compound families.

The synthesis of secondary metabolites depends on the nutrient conditions in the cell. The key elements of life are macronutrients (C, H, O, P and S), major cations (K, Mg and Ca) and micronutrients (metal ions). In *Streptomyces*, complex pathway-specific regulatory mechanisms influence the transition from primary to secondary metabolism in response to environmental conditions, such as temperature, pH, oxygenation and nutrient source availability. Actinobacteria are faced with large changes in the sources of oxygen, carbon, nitrogen, phosphate, sulfur, iron and other nutrients. This requires a strict control of primary and secondary metabolisms to avoid imbalances that could have undesirable consequences for cell growth and secondary metabolite biosynthesis [95,96].

The regulation of secondary metabolism in *Streptomyces* is complex. It is coordinated at different levels and requires extra- and intracellular effector molecules as the central components of the global and cluster-specific regulatory cascades. The transition from primary to secondary metabolism is connected to the morphological and physiological differentiation of *Streptomyces*. The precursor supply is also regulated in the cell through reduction of phosphate levels, catabolite repression and stringent control via ppGpp. It has been demonstrated that low concentrations of phosphate as well as a lack of amino acids, which is sensed by the effector molecule ppGpp, can act as a trigger for secondary metabolite production [97]. Moreover, an interconnection between elevated levels of cAMP (cyclic adenosine monophosphate) and the increased production of secondary metabolites, such as antibiotics and other biologically active substances, has been shown [31,32]. γ-butyrolactones, which are effector molecules, are also involved in the regulation of secondary metabolism and morphological differentiation [98]. In addition to the global regulatory mechanisms, there is biosynthetic cluster-specific regulation. Specific regulatory proteins can activate the transcription of the genes of the associated gene clusters [45,99].

Enzymes involved in antibiotic synthesis, such as phosphatases, become active when the organism’s growth is in stagnation due to nutrient deficiency. Interestingly, lantibiotic production usually co-occurs with the growth of a producer strain, which has been observed for epidermin, nisin and gallidermin, but not for mersacidin, which is produced only after active growth [100,101].

### 3.2. Supply of Key Precursors Carbon, Nitrogen, Phosphate and Sulfur for Secondary Metabolism

Actinobacteria live mainly under long-term nutrient limitations and in constant competition with other organisms in their respective habitats [6]. These non-motile bacteria have no opportunities to move towards more nutrient-rich ecological niches and therefore, they require multiple mechanisms to adapt to their habitats. They can use a variety of nutrient sources and they possess a variety of adaptation mechanisms for rapid changes in nutrient availability [100]. The influence of carbon, nitrogen, phosphate and sulfate sources on secondary metabolite production are well-recognized. The synthesis of the secondary metabolites depends primarily on the provision of building blocks that provide the key elements.

#### 3.2.1. Supply of Carbon for Secondary Metabolism

Carbon can be introduced into diverse compounds as a part of amino acid building blocks or via sugars and their derivatives. The amino acid metabolism building blocks are supplied for the synthesis of secondary metabolites, for example, non-ribosomally synthesized peptides (such as glycopeptides), and ribosomally synthesized and post-translationally modified peptides (RIPPs). From the special synthesis of non-proteinogenic amino acids, which are derived from the precursor synthesis of other amino acids, e.g., via the shikimate pathway, building blocks can also be supplied for the production of secondary metabolites, e.g., glycopeptides. The use of these building blocks in the special synthesis of proteinogenic amino acids was also reported, e.g., pipecolic acid synthesis from lysine [93].

Amino acids synthesized through Acyl-CoA are the building blocks for peptides, proteins and lipids, and can form precursors for diverse metabolites, e.g., leucine, isoleucine, lysine, and valine serve as precursors for industrially important polyketides [102]. Acyl-CoA can be supplied from glycolysis or fatty acid degradation as building blocks for polyketide synthases (PKSs), such as malonyl-CoA. Lipid components for secondary metabolism can be supplied by PKSs. Once absorbed into the cell, amino acids are predominantly metabolized as precursors and are not directly incorporated into antibiotics. It has been shown that the effect of the addition of amino acids to secondary metabolite production is predominantly based on its function as a carbon source [103].

Sugars were described to be building blocks for the production of diverse antibiotics. Metabolic pathways like glycolysis and the pentose phosphate pathway provide sugar phosphates that can be used as building blocks in secondary metabolite biosynthesis [104]. In addition, many secondary metabolite processes take advantage of products or intermediates of cell envelope biosynthesis. Such sugars can either be directly used or they are further specifically modified in the course of secondary metabolite biosynthesis. They can build the backbone structure of antibiotics (e.g., in aminoglycosides). Furthermore, such sugars are used to decorate polyketides and peptides.

The amino acid biosynthetic pathways that involve feedback inhibition are the synthesis pathways for arginine, lysine and aromatic amino acids. Lysine is a building block for many secondary metabolites. It is frequently used as a media additive in industry. The gene encoding the lysine cyclodeaminase is localized in associated biosynthetic gene clusters, like in the cluster of the streptogramin pristinamycin (*pipA*) [105], the lipopeptide antibiotic friulimicin (*pip*) [106], tacrolimus (*fkbL*) [93] and rapamycin [107]. It has been demonstrated for all producers that the overexpression of lysine cyclodeaminase genes leads to an increase in titers of target secondary metabolites. Increased lysine availability has been demonstrated to positively impact and lead to increased secondary metabolite production from cephamycin biosynthesis in *S. clavuligerus* [108] and tacrolimus (FK-506) biosynthesis in *S. tsukubaensis* [93].

#### 3.2.2. Supply of Nitrogen for Secondary Metabolism

Nitrogen can be incorporated into secondary metabolism in different ways via the metabolism of nitrogen-containing compounds, such ammonium, nitrate, amino acids, amino sugars, urea and others. Antibiotic production in bacteria is largely affected by the availability of nitrogen sources, such as ammonium, nitrate, amino acids (e.g., glutamate, glutamine, lysine and serine) and polyamines (putrescine, spermidine, spermine and cadaverine) that come mainly from primary metabolism [8,57]. Nitrogen-containing compounds may increase, decrease, or even stop the production of secondary metabolites. On the other hand, secondary metabolism is also controlled by diverse transcriptional regulators in response to nitrogen availability, e.g., ArgR, AfsQ1, Crp, DasR, PhoP and the response regulator MtrA (master transcriptional regulator A) [5].

Nitrogen-containing compounds such as ammonium can inhibit antibiotic production. On the other hand, nitrate can stimulate rifamycin synthesis. The effect of intracellular nitrogen conditions on antibiotic production depends on the amount of phosphate or carbon in the cell.

Nitrogen can be directly incorporated into secondary metabolites as a part of amino acids, e.g., the amino acids leucine, isoleucine, lysine and valine, which serve as precursors for polyketides. Enzymatic reactions from cellular metabolism, which involve amino acids, can provide reaction products, which are compounds that can provide nitrogen for secondary metabolism as well, e.g., [S,S]-ethylenediamine-disuccinic acid (EDDS) [109]. Another source of nitrogen is aminated carbohydrates (e.g., amino sugars) that can be produced in the direct transamination of sugars. N-O compounds that are provided from specific pathways can be incorporated into secondary metabolism as well, e.g., cremomycin biosynthesis.

#### 3.2.3. Supply of Phosphate for Secondary Metabolism

Phosphate (inorganic phosphate PO_4_^3−^) plays essential roles in cells, such as maintaining the structure of DNA and RNA, cell membrane composition, ATP formation, etc. [23,54]. It is necessary for the synthesis of the main macromolecules of the cell, such as nucleic acids and phospholipids. Phosphate plays signaling roles via phospho-transfer reactions. Furthermore, it is a constituent of high-energy molecules, such as ATP, GTP and polyphosphate, which are essential for most metabolic processes [54,110]. Phosphate is necessary for the initial primary phase of growth, and its depletion may become a limiting component for growth, transition into the stationary phase and the start of antibiotic production. The initial concentrations of phosphate are also critical: in the initial medium, an excess of phosphate drives a higher biomass production and a larger mycelium formation. At the same time, the stationary phase is delayed and the production of a large number of antibiotics is inhibited [100,101,102,103,104,105,106,107,108,109,110,111]. For example, a 4.6 mM phosphate concentration was shown to support of the growth of *S. coelicolor* A3(2) M145 and to boost antibiotic production. In combination with glucose, this provides sufficient energy and reducing power to maintain a high growth rate [111]. Phosphate is found as a linear polymer of variable length, from ten to hundreds of phosphate molecules linked by high-energy phosphoanhydride bonds, which are essential for bacterial survival and adaptation to stress. Bacteria have evolved mechanisms to sequester and store phosphate. The cellular adaptations to phosphate availability are controlled by the two-component system PhoR–PhoP. The genes controlled by this system include genes involved in nitrogen intake, oxidative phosphorylation, nucleotide biosynthesis, glycogen catabolism, antibiotic biosynthesis and morphological differentiation. PhoP is a master regulator since it activates phosphate scavenging, transport, storage and mobilization [26].

Phosphate and phosphate-containing compounds are important precursors of secondary metabolites, providing building blocks for compounds containing phosphate, e.g., fosfomycin isolated from *Streptomyces fradiae* (ATCC 21096) [112] and phosphinothricin tripeptide (PTT) from *Streptomyces viridochromogenes* [113]. The essential precursor for the fosfomycin biosynthesis is the phosphate-containing compound phosphonate, and for the PTT biosynthesis, it is phosphoenolpyruvate (PEP). Phosphonate/phosphate transport systems are common in *Streptomyces* species and are encoded by *phnCDE* genes. Phosphonates utilization also requires the *phnCDE* genes, which include those that code for the subunits of C–P lyase [48]. Phosphate precursor supplementation coupled with medium optimization has been demonstrated to be a wise strategy for improving the yield of antibiotics, e.g., the cyclic lipopeptide antibiotic daptomycin produced by *Streptomyces roseosporus* [114].

#### 3.2.4. Supply of Sulfur for Secondary Metabolism

Sulfur is an important building block for secondary metabolites. The oxidized form of sulfur is present as a sulfuryl moiety (−SO^3−^) that can transform amines in proteins, polysaccharides and lipids. Inorganic sulfate (SO_4_^2−^) can be taken up by active transport across the cell membrane, e.g., by the CysTWA SubI ABC transporter complex in mycobacteria. In further steps, sulfate can be assimilated. For instance, in mycobacteria, this process involves ATP sulfurylase (encoded by *cysND*) or APS kinase (encoded by *cysC*) [115].

The transfer of -SO^3−^ to hydroxyl or amino groups of biomolecules is crucial for the regulation of cell–cell communication and metabolism. Sulfite can be reduced to sulfide (S^2−^) by sulfite reductase (encoded by *nirA*), which is used for the biosynthesis of the sulfur-containing metabolites cysteine, methionine, coenzymes and mycothiol [59]. Sulfur availability has been demonstrated to be important for the methylation reactions catalyzed by SAM. Sulfur can occur in some secondary metabolites, e.g., in lantibiotics, and as a component of the amino acids cysteine and methionine [115].

In *Streptomyces* spp., sulfotransferase enzymes, which transfer a sulfate group onto an acceptor molecule, have been shown to be responsible for the formation of sulfated liponucleoside antibiotics [116]. Mycolithiol has been found in all Actinobacteria and it regulates the cellular redox status and is thus essential for *M. tuberculosis* survival [117]. Furthermore, reduced sulfur-containing metabolite coenzyme A (CoA) is required for lipid and cell wall metabolism [59].

## 4. Conclusions

The natural habitat for Actinobacteria is the soil, which is a very competitive environment with very variable C, N, P and S concentrations. Usually, nutrients are present in low concentrations in soil and are a limiting factor. However, depending on the soil type, season and climate, the nutrient availability in soil may vary ranging from nutrient-poor to nutrient-rich conditions. Due to metabolic potential of *Streptomyces* spp., they can produce metabolites under a wide variety of conditions and are therefore less demanding when cultivated (compared to, e.g., *E. coli*). The study of new industrial processes for antibiotic production should consider multidisciplinary approaches, which can be based on the design of metabolic, physiological and fermentation process aspects. The heterologous expression of genes in microbial hosts has become a common practice to obtain cells with improved fitness, combined with versatile enzymes, structure-guided engineering and directed evolution approaches. Research on new *Streptomyces* strains that produce unknown antibiotics and secondary metabolites has been linked to studies aiming to better understand the morphology, physiology, metabolic changes and signaling occurring during the switch between primary and secondary metabolisms, as well as the supply of precursors from primary metabolism. Cell immobilization in bioreactors with innovative fermentation processes can assure gram-scale production of drugs. Other approaches, such as mutagenesis and genomics, provide the possibility to modify biosynthetic gene clusters in *Streptomyces* spp. to specifically produce high-value antibiotics and other secondary metabolites.
